# Ovarian Fetiform Teratoma in 38 Years Nulliparous Woman: A Case Report

**DOI:** 10.1002/ccr3.70029

**Published:** 2024-12-25

**Authors:** Desalew Gedamu, Mekitie Wondafrash, Meron Berhanu

**Affiliations:** ^1^ Department of Pathology St Paul's Hospital Millennium Medical College Addis Ababa Ethiopia; ^2^ St. Paul's Institute for Reproductive Health and Rights Addis Ababa Ethiopia; ^3^ Department of Pathology Gondar Medical College Addis Ababa Ethiopia

**Keywords:** ectopic pregnancy, Fetiform teratoma, fetus in fetu, homunculus, parthenogenesis

## Abstract

Fetiform teratoma, another name for homunculus, is a rare form of mature teratoma that is highly differentiated and has parts that resemble a malformed fetus. We reported a case of ovarian Fetiform teratoma in a 38 years old nulliparous woman presented with right side abdominal distention of 10 years duration. An ultrasound revealed a heterogeneous pelvic cystic mass that ranged in appearance from fully hyperechoic to fully hypoechoic, suggesting mature cystic teratoma. The patient underwent laparotomy. Histopathology of Cystectomy specimen was found to be compatible with Fetiform teratoma. It is important to distinguish this highly differentiated benign neoplasm with potential somatic malignant transformation from Fetus in fetu and ectopic pregnancy. To establish the diagnosis, a thorough clinicopathologic examination, laboratory tests for pregnancy and relevant cytogenetic tests for Zygosity should be performed.

**Summary**

- Fetiform teratomas are uncommon highly differentiated mature teratomas that have parts that resemble malformed fetuses, and believed to result from parthenogenic development of primordial germ cells.
- In this case there a unilocular right pelvic cystic mass contained numerous small bones of appendicular and axial skeleton lacking vertebral column.

## 1 Introduction

Teratoma is a germ cell tumor composed of mature tissues derived from the three embryonic germ layers (ectoderm, mesoderm, and endoderm). Most ovarian teratomas are mature and common benign tumors in women of reproductive age group presented as pelvic or lower abdominal mass.

Fetiform teratomas are uncommon mature teratomas that are highly differentiated and have parts that resemble malformed fetuses. The Latin term homunculus, which means “Little man,” can be used interchangeably. Mature cystic teratomas are believed to result from parthenogenic development of primordial germ cells, which are homozygous after completing the first meiotic division [1, 2]. This must be distinguished from ectopic pregnancy and fetus in fetu (which are congenital abnormalities of parasitic twin in diamniotic monochorionic pregnancy that are typically found inside the body of a newborn or the infant's body). Here we report a case of fetiform teratoma revealing unilocular cystic cavity contained well developed very small bones lacking vertebral axis.

## 2 Case History/Examination

A 38‐year‐old nulliparous woman presented with a 10‐year history of right side abdominal distention. She started to have pain 5 months prior to hospitalization. There was no notable surgical or medical history. She had no past history of amenorrhea and her menstrual cycles were regular. A mobile, non‐tender, round, and firm mass was found in the right pelvic area during an abdominal examination.

An abdominal ultrasound revealed a 17 × 15 cm right pelvic cystic mass that appeared heterogeneous, ranging from fully hypoechoic to completely hyperechoic. A level of fat‐fluid was detected in the cystic mass. It was possible to see multiple linear hyperechogenic interfaces floating inside the cyst. Additionally, there were numerous linear echogenic foci with distinct acoustic shadowing. The preoperative results collectively indicated a mature cystic teratoma. The range of the laboratory results was normal.

## 3 Methods (Differential Diagnosis, Investigation, and Treatment)

A laparotomy was performed with a clinical impression of a cystic ovarian tumor after appropriate investigation. A cystic mass adherent to the intestine, omentum, and mesentery was discovered during surgery. It was apparently encapsulated and released out of its adherence with ease. On the fourth postoperative day, the patient was discharged with a smooth clinical outcome.

**FIGURE 1:**
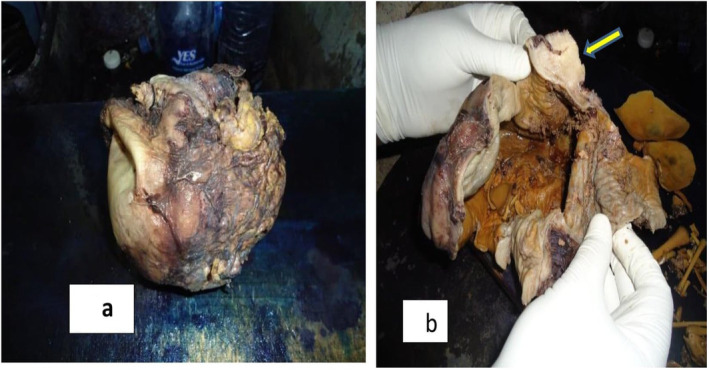
Formalin fixed surgically removed pelvic cystic mass 18 × 15 × 10 cm: (a) pre‐opened up external surface. (b) Appearance of inner part of the cyst. The inner surface is grayish and wrinkled with focal mural gray white solid nodule 2 × 2.5 × 3cm (yellow arrow). Thick yellowish greasy intracystic content has been observed.

**FIGURE 2:**
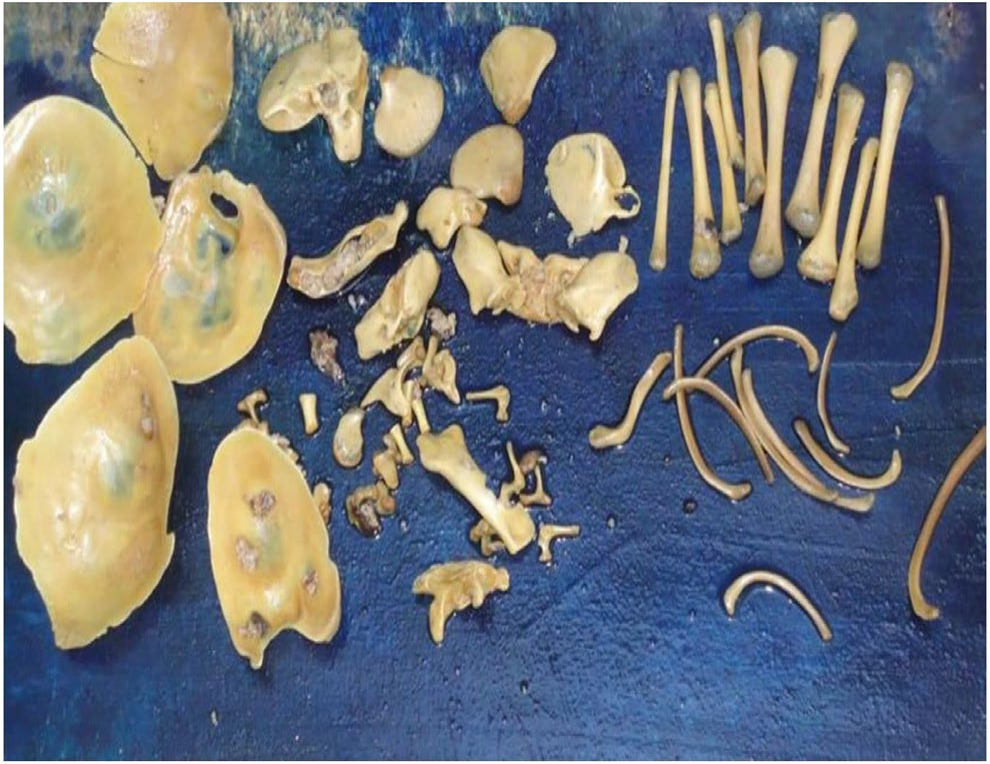
Gross appearance of numerous small sized well‐formed bones evacuated from the cyst cavity. Flat bones of skull (left), bones of scapula, pelvic girdle and small tubular ones (middle) and numerous long and rib bones (right).

After the mass was properly fixed in 10% formalin, a gross evaluation revealed an 18 × 15 × 10 cm partially capsulated, rough‐surfaced, pale yellowish cystic mass (Figure 1a). When opened, it revealed to be a unilocular cyst with a 0.3–0.5 cm wall thickness and had a corrugated, smooth, grayish white interior surface. The inner surface was found to be lubricated by an oily and Greasy substance. A single, 2 × 2.5 × 3 cm, solid, grayish nodule was present (Figure 1b). Many small and well‐formed bones, including a set of appendicular skeleton, flat bones of pelvic girdle, skull and ribs were found inside the cyst cavity (Figure 2). The largest bone measured 4.5 cm in length, while the flat bones' approximate concavity diameter measured 3 cm. The vertebral column's bones, however, were not depicted.

**FIGURE 3:**
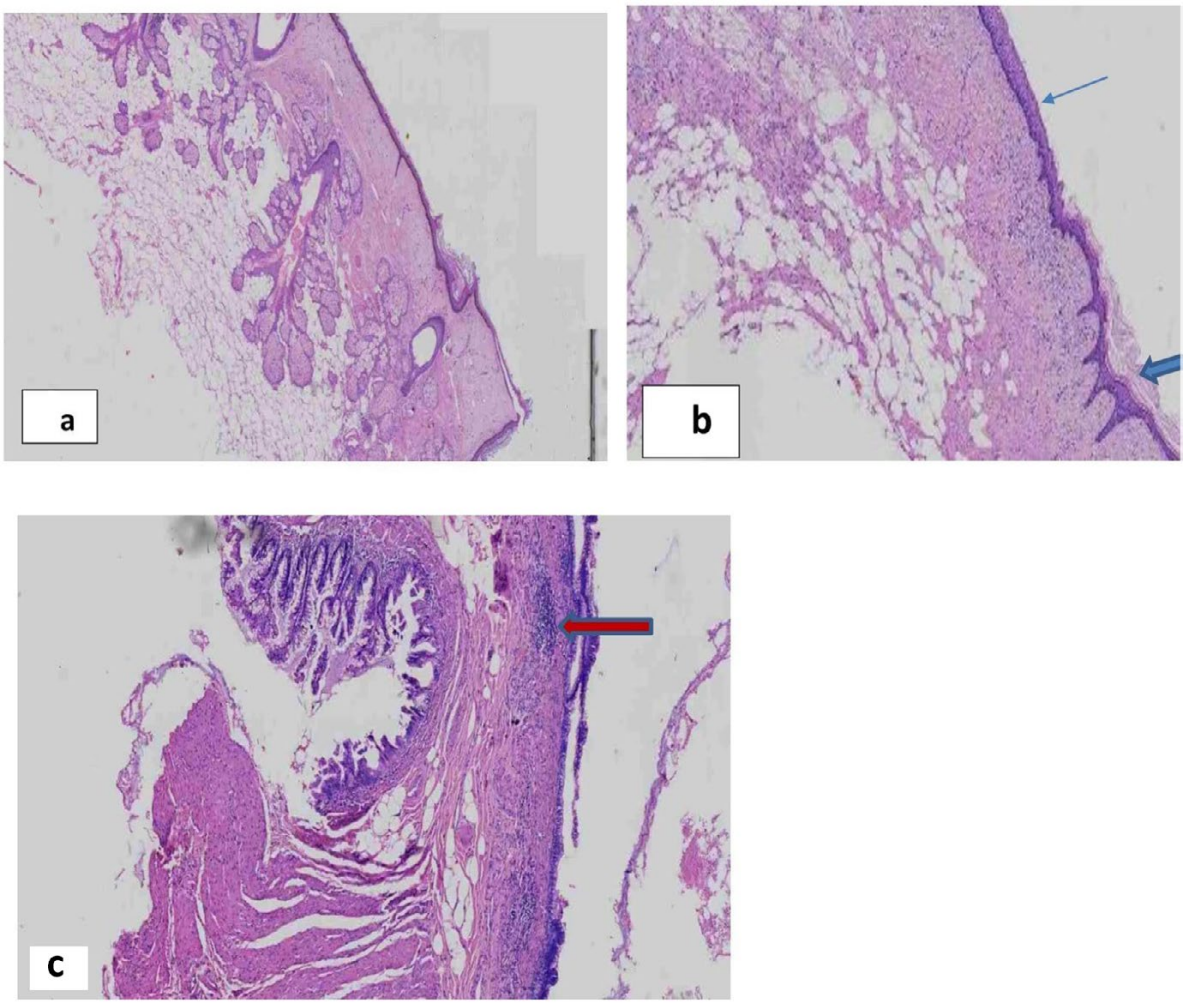
Photomicrograph of parts resected cystic wall: (a) Overlying squamous epithelium and skin adnexal structures (sebaceous glands and hair follicles). Hematoxylin–eosin, original magnification ×4 objective. (b) Keratinized (thick arrow) and non‐keratinized (thin arrow) squamous epithelium with subcutaneous fat. Hematoxylin–eosin, original magnification ×10 objective). (c) Intestinal tissue with lymphoid nodules (red arrow). Hematoxylin–eosin, original magnification ×10 objective).

Under a microscope, the cyst lining revealed squamous epithelium that was both keratinized (thick arrow) and non‐keratinized (thin arrow) with underlying fibro ‐adipose tissue and skin adnexal structures (Figure 3a,b). There is an entire layer of intestinal tissue in one area of the wall with lymphoid aggregates (Figure 3c). It is important to differentiate this extremely specialized tumor from ectopic ovarian pregnancy and fetus in fetu. The axial skeleton of a fetus in fetu is highly developed and segmented (post primitive streak development), and other organs must be placed appropriately around it.

## 4 Conclusions and Results

As there was no history of amenorrhea in the index case, ectopic pregnancy could be easily ruled out. The absence of the visceral tissue and vertebral body, as well as the nature of histologic appearance of epithelial lining, was more consistent with fetiform teratoma.

### 4.1 Outcome and Follow‐Up

The patient was discharged with smooth postoperative course after 4 days of hospital stay. However, she was lost for follow up for her residency was deep in rural community with inaccessible contact address. However, it would have an impact on understanding the general condition of the patient and prognosis in particular.

## 5 Discussion

Usually, the testis or ovaries are the source of fetiform teratomas. Although the age distribution is comparable to that of conventional teratomas, reports have indicated that cases have been reported from 3 months of age to 65 years of age [1, 3]. In nearly all reported cases, the fetiform structures were skin covered with hair and skin adnexal structures. A common finding was rudimentary upper and lower limbs with occasional digits which lack well‐formed vertebral axis. Nonetheless, as noted in Joutel et al. [1], the homunculus possessed a fully formed axial skeleton in addition to numerous other structures, such as the head, trunk, brain, eyeball structure, and trachea.

Our case was distinct because it did not reveal a fetiform mass covered in skin. Every skeletal component, with the exception of the vertebral body, seemed to be grossly identifiable inside the cyst cavity, where the cyst wall functioned as a bag. It is important to differentiate this extremely specialized tumor from ectopic ovarian pregnancy and fetus in fetu. The axial skeleton of a fetus in fetu is highly developed and segmented (post primitive streak development), and other organs must be placed appropriately around it. This is how most authors agree that fetus‐in‐fetu differs from teratoma [3, 4, 5].

The genetic makeup of the fetus in fetu and the host are identical and heterozygous in contrast to the homozygous composition of teratoma. Clinically, fetus in fetu has been identified in the early stages of life and in infancy; extremely rare cases have been reported in individuals over the age of 15 [4]. Seventy percent of cases present as an abdominal mass, and no cases within the ovary have been documented. It is found in the upper retroperitoneum in 80% of the time [1, 4, 5]. In contrast, fetiform teratoma is most frequently found in women of reproductive age who present as an ovarian mass in the pelvic region. Placenta, trophoblastic tissue, and chorionic villi should be identified and show positive test results for beta human chorionic gonadotrophic hormone (**β‐HCG**) in cases of ectopic pregnancy. In our instance, a set of long bones, as well as skull and pelvic girdle bones, created confusion to categorize the case. In conclusion, based on degree of organization, it is not easy to distinguish fetiform teratoma from fetus in fetu and ectopic pregnancy. As a result, a complete pathologic examination, a careful clinical history, laboratory test and cytogenetic studies for zygosity are necessary for a definitive diagnosis. Limitation of this report includes lack of genetic testing with potential impact on patient prognosis.

## Author Contributions

**Desalew Gedamu:** conceptualization, data curation, methodology, project administration, writing – original draft, writing – review and editing. **Mekitie Wondafrash:** supervision, writing – original draft, writing – review and editing. **Meron Berhanu:** supervision, writing – original draft, writing – review and editing.

## Ethics Statement

Ethical clearance was obtained from the Institutional Review Board of St. Paul's Hospital Millennium Medical College. Confidentiality of patient's records was kept and no patient information was transferred to any other organ.

## Consent

Written informed consent was obtained from the patient for publication.

## Conflicts of Interest

The authors declare no conflicts of interest.

## Data Availability

Data can be provided if needed.
